# Factors influencing pharmaceutical pricing - a scoping review of academic literature in health science

**DOI:** 10.1186/s40545-019-0183-0

**Published:** 2019-06-27

**Authors:** Maria Angelica Borges dos Santos, Leticia Lucia dos Santos Dias, Cláudia Du Bocage Santos Pinto, Rondineli Mendes da Silva, Claudia Garcia Serpa Osorio-de-Castro

**Affiliations:** 1 Vice Direção de Escola de Governo, Escola Nacional de Saúde Pública Sérgio Arouca, Fundação Oswaldo Cruz, Av. Brasil, 4036 sala 902, Manguinhos, Rio de Janeiro, Brazil; 2 Fundação Oswaldo Cruz, Rio de Janeiro, Brazil; 3 Instituto Integrado de Saúde da Universidade Federal de Mato Grosso do Sul (INISA/UFMS), Campo Grande, Brazil; 4 Departamento de Política de Medicamentos e Assistência Farmacêutica da Escola Nacional de Saúde Pública Sergio Arouca, Fundação Oswaldo Cruz, Rio de Janeiro, Brazil

**Keywords:** Medicine price, Government regulation, Review literature as topic

## Abstract

**Introduction:**

Pharmaceutical pricing has only recently gained space in mainstream health science literature.

**Objectives:**

Bibliometric and content description of health science academic literature and ad hoc analysis of grey literature on factors influencing pharmaceutical pricing on databases commonly accessed by healthcare professionals.

**Methods:**

Scoping study with no time limits performed in Medline, Scopus and Scielo, and relevant sites and databases for grey literature, using search terms with database-appropriate keywords**.**

**Results:**

Two hundred four articles were published in 103 peer-reviewed journals between 1981 and 2016 (last search year). In grey literature 78 documents were retrieved in the final selection. Five key thematic clusters for analysing pharmaceutical pricing emerged: market dynamics, segmented into (i) supply-related, (ii) consumer-related and (iii) product-related; (iv) trading strategies, either buyer’s or seller’s and (v) regulatory approach. In peer-reviewed literature there is an overall dominance of themes referring to trading strategies and regulatory approaches and a wide thematic cluster scope. Over half of this literature was produced after the year 2010. International agency technical papers make up the most significant contributions of grey literature, with a clear focus on regulatory approaches to pricing and wider consideration of emerging countries. Research lags in the literature on factors affecting pharmaceutical pricing include impacts of financing schemes, market liberalization, internet trading and biosimilars on prices, with insufficient discussion identified for the effects of discounts/rebates, profits and price transparency.

**Conclusions:**

Interest in pharmaceutical pricing literature is increasing. Robust evidence-producing study designs for pricing interventions will be a welcome development.

**Electronic supplementary material:**

The online version of this article (10.1186/s40545-019-0183-0) contains supplementary material, which is available to authorized users.

## Background

In simple economic terms, prices are the monetary worth established for a product during a transaction between economic agents (producers, distributors, consumers and regulators). Agent perception of product value and equilibrium between product supply and demand are both paramount for price definition [1, 2].

Pharmaceuticals are much-valued goods among the many traded goods in contemporary societies. Factors influencing their pricing are relevant both to the welfare and to the economic development of nations. On one hand, prices influence medicines’ affordability and access to health products and, on the other, they are a crucial incentive for pharmaceutical companies to develop new products and, thus, for industrial policy [3]. Initial studies on the topic were developed outside health science academia and published mainly in business, industrial economics and law academic journals.

This older literature on the pharmaceutical market dates back from the beginning of the twentieth Century and involves substantial publication in the form of books. In the 1940s, it depicts the ongoing discussion on advertising and the medical classes [4], distinct advertising strategies [5] and levels of advertising activity in different markets [6], reflecting a focus on strategies for influencing value perception.

The initial theses concerning pharmaceutical industry pricing behaviour involving supply and demand in a stricter fashion followed a more general discussion on industrial economics. Specific aspects of the drug industry pricing behaviour, focusing on supply-related factors (industry profits and anticompetitive practices), were brought out in the Kefauver hearings in the early sixties, together with a questioning of patenting rules and a demand for proof of efficacy [7]. Much of the literature in this period examined supply-side and demand-side market dynamics [2], highlighting that, where only one or a few firms sold particular products, prices had a tendency not to vary widely. Specific examples of this were shown for antibiotics and corticosteroids [8].

According to these initial discussions, the pharmaceutical industry’s behaviour relative to pricing fitted, at the macrolevel, into the administrative price thesis [9, 10], which takes into account the oligopolistic nature of the pharmaceutical industry [11]. Barriers to entry linked to high investments usually required to start and run a pharmaceutical company further enhance their market power [12].

Market equilibrium conditions and the essentiality of products in this specific economic sector set the stage for the introduction of governmental regulation. Regulation comprises various strategies to influence prices through the creation of rules impacting supply-related and demand-related market-dynamics [13]. It also includes policies aimed at product-related market dynamics, which are well illustrated by the effects of patents and generic entry [14] on the prices of pharmaceuticals [15].

Discussions on regulation and phenomena linked to pharmaceutical pricing have gained considerable space in mainstream health science literature in the last thirty years. A seminal discussion was posed by Backhaus in 1986 on the impacts of regulation on innovation and competition [16]. Specific regulatory approaches, such as internal and external reference pricing [17], came to be discussed along the years. Criteria for pharmaceutical price-setting based on economic evaluation [18] emerged in the nineties, following the release of pioneering draft guidelines by Australia in 1990 [19]. The concept of aligning prices to therapeutic results, known as value-based pricing [20], seems to be the latest development, thriving on both economic evaluation and on the earlier Kefauer quest for proof of efficacy [7].

As prescribers and stakeholders, health care workers and managers increasingly come upon discussions surrounding the broader topic of health economics, including medicine pricing. To follow this debate is not, however, a simple endeavour, as economic concepts set the stage. In addition to influential health international agencies publications, academic literature in one’s own field is a natural reference in this case. An overview of what is being discussed can be interesting both as a starting point to those planning to develop a deeper understanding of the theme and to specialists working on specific research in this area.

With this is mind, we set off to revise academic health science journals and “grey literature” (especially international health and development agencies publications) on pricing of pharmaceuticals most readily accessed by health care professionals. As we are trying “examine the extent, range, and nature of a research activity”, we opted to conduct a scoping study [21]. This would allow us to synthesize knowledge in an exploratory manner, mapping key concepts, origins and types of evidence [22].

Specifically, this scoping study aims to answer the following questions: (i) Which countries, journals and author affiliations dominate this field in academic production? (ii) What kinds of study design are more frequent? (iii) What are the key concepts and themes brought up by the literature over time? (iv) What are the key differences between what is being offered in academic and grey literature databases on the topic? (iv) What are the knowledge gaps in this literature?

## Methods

This is a scoping review of studies on factors affecting prices found in databases routinely assessed by health care professionals and managers. An initial study protocol, available at Arca Fiocruz [23], had academic (“peer-reviewed”) literature most readily accessed by health care professionals as focus. To broaden the overview of available production, a post-hoc analysis of grey literature, with specific attention to databases of international healthcare and development agencies, was eventually carried out.

We drew upon theories of demand and supply in differentiated products markets and regulation theories [1, 24, 25] to tentatively elect supply, demand, product, regulation and trading (transaction) strategies as key elements to hold categories to conceptualize the reviewed literature. For pricing category identification, we drew upon the broad historical literature review presented in the preceding background section and on Kina & Wosinska [26].

Data supporting manuscript results is available upon request to authors and at Arca Fiocruz [23]. To guide analysis, a framework for analysis was built (Table 1) and methodological steps are presented below.

**Table 1:** Scoping review framework

	Aspects
Charting dimensions (studies)	
Geographic localization and author affiliations	1) Country where study was developed 2) Region/Country where study was published 3) Author affiliations (by type) 4) Journal name
Study designs and timeframe	1) Year of publication 2) Study design
Thematic cluster categories	1) Supply related market dynamics 2) Demand related market dynamics 3) Product related market dynamics 4) Trading strategies 5) Regulatory approaches
Charting dimensions specific to grey literature	
Origin	1) WHO 2) OECD 3) World Bank 4) Other grey literature
Timeframes and publication type	1) Year of publication 2) Type of publication (report, working paper, discussion paper, series)

### Part 1. Producing the bibliometric database

#### Step 1 – literature search

To balance feasibility with breadth and comprehensiveness [21] we searched MEDLINE (via PUBMED), Scopus and Scielo (via BVS) databases for peer-reviewed papers on factors influencing medicine prices. Period covered was since inception up to 2016 and the search was limited to published papers. Initial search was performed in May, 2016 and revised in November, 2016, when the research team agreed on further refinements of inclusion/exclusion criteria. Controlled vocabulary, as well as text wording were both employed. Full electronic search strategy for Pubmed was (((((“price”[Title] OR “payment”[Title] OR “pricing”[Title] OR “cost” [Title] OR “regulation” [Title] OR “government regulation”[MeSH Terms]) AND ((“medicines”[Title]) OR “pharmaceutical preparations”[MeSH Terms]) AND “humans”[MeSH Terms])))), with adaptions for use in the other databases. Neither reference list nor hand searching were conducted.

Grey literature search was done separately, in March 2018. We initially reincluded 263 off-category (not an article) publications from the peer-reviewed literature. We then went on to search the Grey Literature database and websites of the World Bank, World Health Organization (WHO), European Union, Organization for Economic Cooperation and Development (OECD) and National Academy Press. Search strategies included a combination of the terms “Medicines” (title), “price” or “pricing”, “payment” or “cost” and “regulation” in title or keywords and variations thereof, adapted for suitability to individual databases. For grey literature and international agency documents, we eventually conducted some hand-searching when a book (for other relevant chapters) or publication series (for other publications of the same series) was identified. A detailed description of search strategies syntaxes is provided in Additional file 1: Table S1.

#### Step 2. Manuscript selection based on inclusion and exclusion criteria

Titles and abstracts of manuscripts retrieved were independently screened by two reviewers to determine eligibility. Inclusion criteria were: (i) price as the outcome variable, as we were interested in factors affecting prices and not on the effects of prices (ii) presence of English language abstract/summary (iii) availability of complete text (iv) presence of the word ‘price’ in title, abstract or summaries. Exclusion criteria were: (i) duplications (ii) off-topic citations.

Disagreements or doubts were resolved by discussion with the full team of authors and, when unresolved, the full text was for clarification. A quality assessment of the studies was not carried out.

#### Step 3. Charting bibliometric data and defining study designs

Results were charted by two independent researchers using a previously designed structured spreadsheet that had been tested by the team. Initially the title and abstract title were extracted. Study characteristics included: journal/source and year of publication, study setting (target country), when applicable; country where study was produced; author affiliation (institution producing the study); and study design/document type.

Defining a study design taxonomy was particularly challenging. Robust designs as far as evidence production is concerned are still unusual [27]. Much of this literature follows the economics tradition, where producing evidence for practical economic approaches has only recently become an issue [28] following a “naturalistic turn”, which stresses a descriptive rather than a prescriptive function for the discipline [29].

In the preface of his “The Theory of Price” [1], George J. Stigler presents a tentative framework for study taxonomy. In line with his reasoning, we divided studies into theoretical and empirical studies and further divided theoretical studies into discursive (logic-based) or mathematical modelling studies (evidence-based).

Empirical studies were characterized as observational studies involving the explicit use of data (legislation, interviews and other documents, as well as datasets). These were further categorised as: (i) descriptive, when neither specific hypotheses were tested nor datasets were used to discuss the object, or (ii) quasi-experimental, devoted to the testing of specific hypotheses, with the use of data-sets and statistical analysis techniques. Macroeconomic studies – such as those on markets and prices–are not suited to experimental designs. However, “natural experiments” occur in the context of policy/economic changes. According to an economic definition, “quasi-experimental” designs would be structurally identical to experimental designs, but preclude interventions performed by the investigator, using observational (datasets, for instance) instead of experimental data [28].

For peer-reviewed literature, all study characteristics were described according to overall frequencies in literature. Countries producing the studies and study designs were further described according to time intervals.

### Part 2. Establishing relevant categories and thematic clusters

#### Step 1. Identifying categories

The main price-formation related concepts addressed were discussed in a series of meetings to allow researchers to gain an overall conceptual, temporal and geographic perspective on issues emerging in literature. Identifying relevant categories and themes is a central part of the charting process in scoping studies [21]. To that end, the lead author reviewed extracted data on spreadsheets and, based on the contents of titles and abstracts, gradually selected concepts (categories) that best characterized the contents of the reviewed literature.

To describe key categories in the literature we resorted to techniques suggested as preliminary steps in thematic analysis [30, 31]. We initially coded the texts based on the frequency of the most relevant terms in titles and abstracts, which convey the main concepts or “categories” guiding these studies. An Excel localizing tool was used to systematically identify categories in the titles and abstracts in selected studies. The context of use of the category/term was checked in each paper to assure that it was in fact related to the intended meaning. Research, for example, was a term that could come up as referring to R&D activity – the intended meaning - or to general research context/academic activity, i.e., “more research on the theme is needed”.

#### Step 2. Valuing categories according to their centrality in studies

Each paper of the peer-reviewed literature was also coded according to “key categories” and “accessory categories” found in the title and abstract. To convey the centrality of categories in each study, categories received score 2, if deemed central in the study, or 1, if deemed accessory. Centrality was defined as the sum of scores divided by the frequency of each category. Scores could range from a minimum of 1, meaning low centrality and representing the fact that the category was never a central topic in studies, to 2, meaning a hypothetical situation in which a category was always a central topic in studies in which it was cited. Each individual manuscript was coded for a minimum of one category, but more than one category was allowed. Also, more than one central category could be identified in any individual study. For grey literature, only “key” categories or thematic clusters were recorded, which in themselves were considered as conveying theme centrality.

Coding was resumed by individual analysis of the categories brought up in each paper. Titles and abstracts or summaries of papers for which initially no category was identified were reanalysed. In this process, new categories were eventually identified, and these were again searched through the whole set of selected papers. Categories were then individually described according to their frequency and score-based centrality. Only categories found in more than five papers/documents were included in the final results.

#### Step 3. Defining thematic clusters and synthesis of results

To define thematic trends in literature, we finally proposed clusters of related categories that best characterized discussion topics along time and named them “thematic clusters”. A theme is a pattern that captures something significant or interesting about the data and/or research question, clustering codes that fit together [30]. To propose clusters, we drew upon theoretical frameworks named in the beginning of the methods section. Team members reviewed themes to confirm the interpretations that had been generated. Any study could be coded in more than one thematic cluster, according to the distinct categories mentioned in it.

Frequency and centrality of these thematic clusters were described based on the aggregate frequency of categories and on weighted-average of category centrality contained in each specific theme. We thus described overall thematic frequency and centrality and further detailed thematic frequency over time.

In the final report, we synthetized results separately for peer-reviewed and grey literature (divided into other “grey literature” and international agency publications) using a narrative approach, tables (timeframe of study design, according to time intervals; category and thematic cluster frequency and centrality) and graphs (spatial and temporal distribution of papers). A last step was to identify key differences between peer-reviewed and grey literature and the knowledge gaps with direct relevance to the scoping review questions.

## Results

**Fig. 1:**
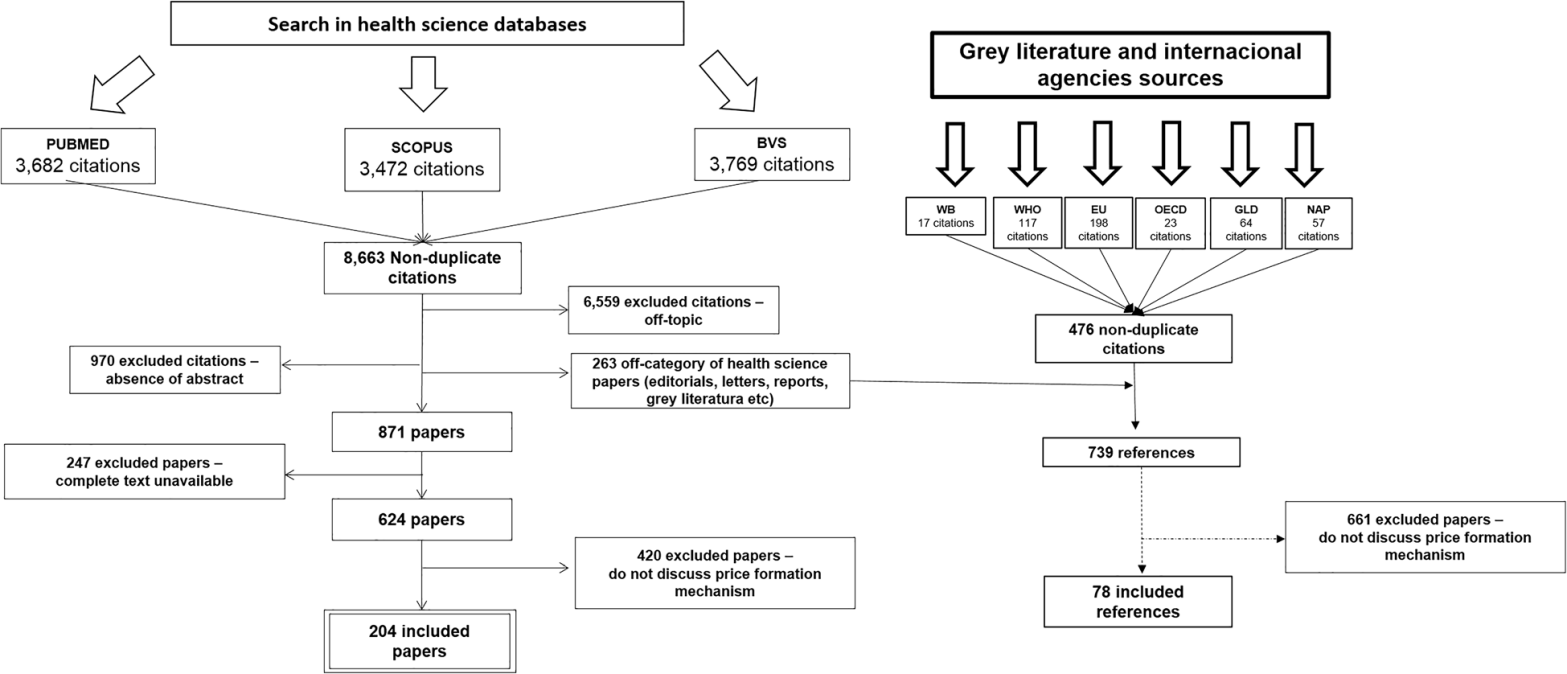
Flowchart

Our search was conducted according to the steps in the flowchart (Fig. 1) and yielded 204 studies, published between 1981 and 2016 (last search year).

### Synthesis of results for peer-reviewed literature

#### Which countries, journals and author affiliations dominate this field of academic production?

The 204 studies were retrieved in 103 journals. Among these, 48 were published in 36 non-health science journals, with the greatest numbers of studies in Applied Economics (*N* = 4), Managerial and Decision Economics (*N* = 3), Journal of Public Economics (*N* = 3) and Journal of Law and Economics (*N* = 3). Four health science journals having health policy and health economics as scope account for one fourth of the overall manuscripts-Health Policy (*N* = 16, 7.8%), PharmacoEconomics (*N* = 14, 6.9%), Journal of Health Economics (*N* = 11, 5.4%) and The European Journal of Health Economics (*N* = 11, 5.4%).

Seventy-four studies (36.3%) were published in journal not indexed by Medline via PubMed. Public health journals published seven (less than 5%) of the retrieved manuscripts. The greatest number of articles (*N* = 110; 53.9%) were published in the 2010s. Also, an increase in publication volume was observed along the study period (Additional file 1: Figure S1). An analysis of the average number of studies produced since 2000 shows a steady rise from 6 studies/year, from 2000 to 2005, to 14.4 studies/year, from 2010 to 2015, and a new peak occurring after this.

Most authors were from academia (*N* = 150, 73.5%), which was the overall largest contributor to the discussion on medicine pricing. Academia was followed by private sector affiliations, highlighting the 2000s and 2010s as the years with the most intense production on the topic, with 73 and 110 publications, respectively (Additional file 1: Table S2).

**Fig. 2:**
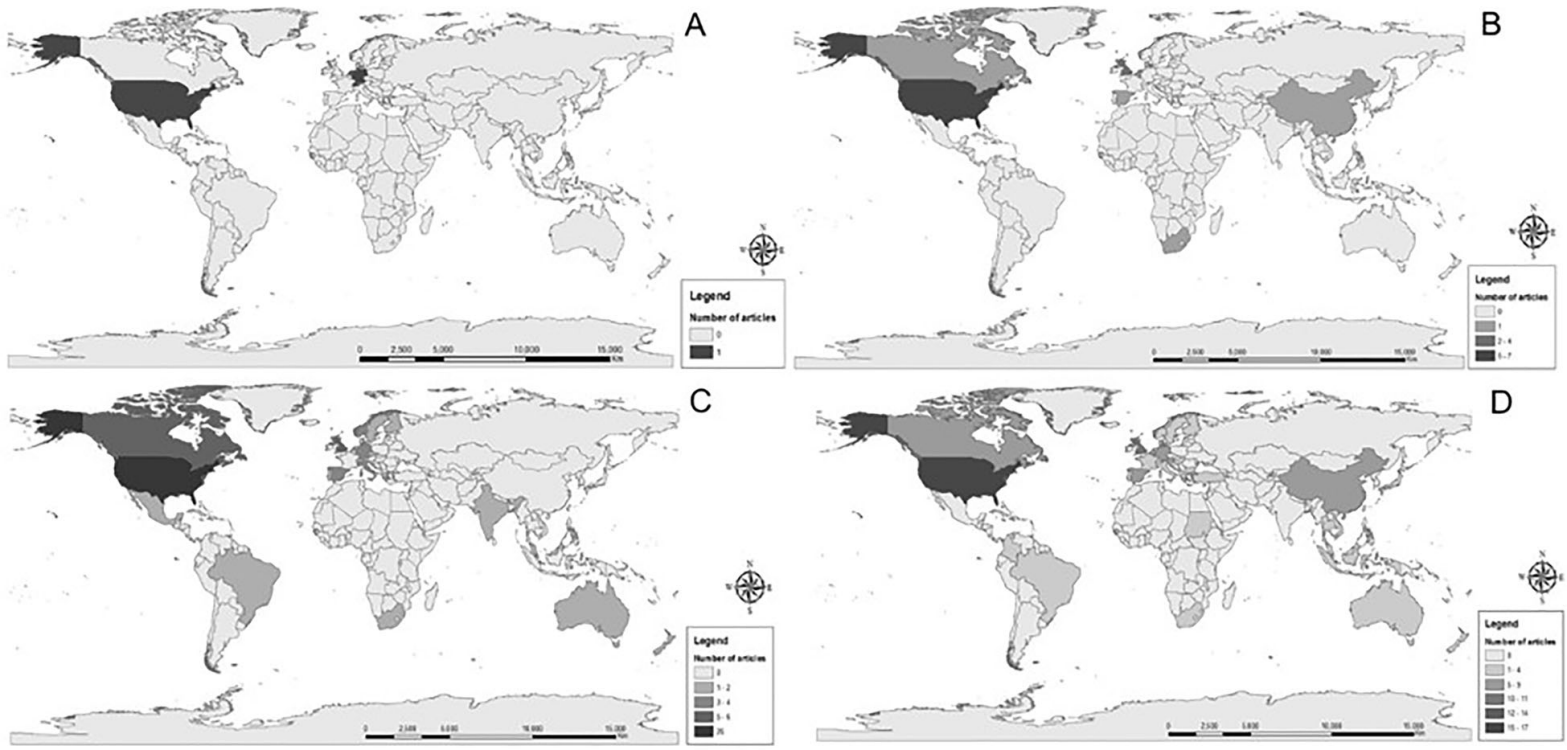
Countries producing articles on pharmaceutical pricing factors indexed in peer-reviewed databases. 1981–2016. **a** 1980–1989; (**b**) 1990–1999; (**c**) 2000–2009; (**d**) ≥ 2010

North American institutions published articles in all decades, always producing the highest number of studies. There was a growing diversification of article origin along the years, meaning that more countries are discussing and publishing in this area (Fig. 2).

#### What kinds of study design are more frequent in peer-reviewed literature?

The most frequent study design was empirical quasi-experimental studies (*N* = 106; 52%) (Additional file 1: Table S3). Quasi-experimental studies were predominantly represented by panel data analyses, based on inferences with data having large cross section and long time series. Empirical descriptive studies followed (*N* = 40; 19.6%), highlighting the overall dominance of empirical studies in this literature. Most of the empirical descriptive studies comprised descriptions of country’s regulatory framework for pharmaceutical pricing.

Theoretical studies (*N* = 58; 28.4%) predominantly discussed concepts in a discursive fashion, but there was still space for theoretical models, as in other fields of economic literature (Additional file 1: Table S3).

Around 80% of the studies (*N* = 162) presented descriptions and discussions relating to one or more countries, which could be described as case studies. Among these, one third referred to European Union countries (*N* = 53; 32.7%), with 8 (4.9%) studies targeting Germany. However, the United States (US) was individually the most studied country (*N* = 22; 13.6%). There was also significant literature on Scandinavian (*N* = 14; 8.6%) and upper-middle-income (*N* = 24; 14.8%) countries, specially China (*N* = 8; 4.9%). Less than 5% were studies specifically targeting individual lower-income countries. Such studies were only seen from 2010 onwards, with 5 featuring lower-middle income countries (Egypt, Indonesia, India, Sudan and Vietnam) and 2 on low-income countries (Mozambique and Mali) (Additional file 1: Table S4).

#### What are the key concepts and themes brought up by the peer-reviewed literature over time?

An initial quantitative analysis of key concepts in this literature, as defined by word frequency in the titles and abstracts of the scoped studies, identified 23 categories (Table 2). Two other categories also emerged - ethics (*N* = 3) and financing schemes for pharmaceuticals (*N* = 5) - but were not deemed sufficiently frequent to be included.

**Table 2:** Category and thematic frequency and centrality in peer-reviewed literature on medicine pricing

Thematic cluster	Category	Category frequency (N)	Category centrality (score)	Thematic frequency^a^ (score)	Thematic centrality^b^ (score)
Supply related market dynamics	Supply chain price components	16	1.56	68	1.38
	Profit	24	1.08		
	Research & development	19	1.42		
	Advertisement/Marketing	9	1.44		
Demand related market dynamics	Demand	24	1.33	90	1.26
	Consumer	33	1.21		
	Population income	21	1.43		
	Price transparency	12	1.08		
Product related market dynamics	Innovation	24	1.27	190	1.34
	Competition	58	1.43		
	Patent	43	1.26		
	Generics	65	1.42		
Trading strategies	Parallel trade	12	1.67	90	1.53
	Firm pricing/launch strategies	36	1.67		
	Tendering/ procurement	13	1.31		
	Rebates/ discount	18	1.11		
	Differential pricing/international price discrimination/tiered pricing	11	1.91		
Regulatory approaches	Value base pricing/	17	1.65	155	1.52
	Profit control/price control	32	1.41		
	(Cost)-effectiveness studies/health technology assessment	20	1.25		
	Internal reference pricing	38	1.55		
	External/international reference pricing	15	1.67		
	Reimbursement policies	33	1.61		
	Averages	25.78	1.42	118.60	1.41

^a^Aggregate frequency of theme-related categories

^b^ Weighted average of category centrality for the theme

Categories were further grouped along the analysis process into five thematic clusters: market dynamics, segmented into three clusters: (i) supply-related, (ii) consumer-related and (iii) product-related; (iv) trading strategies, either related to sellers (“firm pricing strategies” and “rebates/discounts”) and buyers (parallel trade; tendering/procurement; differential pricing and equivalent concepts); and (v) regulatory approaches, segmented into general regulatory policies or specific regulatory strategies (value-based pricing, profit control, cost-effectiveness/economic evaluation, internal and external reference pricing and reimbursement policies) (Table 2).

Categories presenting overall above-average frequencies included competition, generics, firm pricing/launch strategies, internal reference pricing and reimbursement policies. Among the most frequent categories only firm-pricing strategies, internal reference pricing and the closely related category reimbursement policies had very high centrality in studies, meaning they tended to be central topics in studies. Above-average centrality also noted for a number of categories in the trading strategies and regulatory approaches clusters (Table 2).

Two of categories were found to be represented by synonymous or near synonymous terms. These were: differential pricing [32], for which the terms international price discrimination [33] and tiered pricing [34] were also used; and the more recent category value-based pricing [35], also described by the very close categories conditional pricing [36], early-benefit pricing [37], additional-benefit pricing [37], performance-based pricing [38] and pay-for-outcome pricing [39]. Also, a single term could convey more than one meaning. Reference pricing, for instance, could either refer to the use of external or international reference prices or to in-country prices as references for pricing new or specific classes of pharmaceuticals [40] or to reimbursement policies for pharmaceuticals. In this latter context, a reference price stands for a maximum value reimbursed for each drug, above which co-payments by consumers would be in order if they opt for specific and higher-priced brands [41]. The category was thus distinguished into internal and external reference prices.

Following thematic clustering, regulatory approaches showed the greatest centrality, together with trading strategies (Table 2). Also of note are the low frequency of mentions to supply-related market dynamics categories and the low centrality of the theme demand-related market dynamics, meaning that these usually correspond to accessory discussions in the literature.

**Fig. 3:**
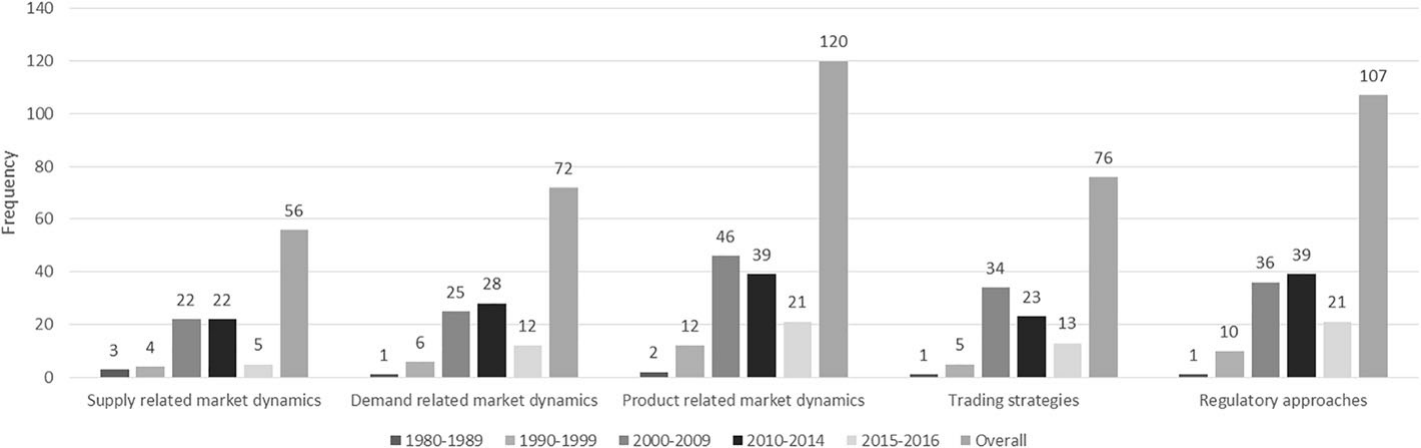
Thematic analysis in peer-reviewed literature. Timeline of thematic frequency (%) in peer-reviewed studies. 1981–2016

Product-related market dynamics and regulatory approaches have the highest overall and current thematic frequency, but there are interesting thematic shifts in specific time intervals (Fig. 3).

### Grey literature and international agency documents synthesis

The final selection comprised 78 documents, of which 39 retrieved in grey literature and the other in international healthcare and development agencies: WHO (29), OECD (7) and World Bank (3). Non-European and non-OECD countries were preferentially targeted in country studies stemming from this literature (36/49) and there is a wide dominance of empirical descriptive (case) studies.

The greater part of the grey literature (27), international agency publications excluded, comprises chapters of 4 books and academic dissertations (5). Thematic scope seems to be as wide as in peer-reviewed literature.

Initial selected documents from international agencies date from 1995. Documents come in various formats (working papers, Technical documents, publications in booklet format and policy briefs) and may be available in several WHO and OECD member country languages. WHO publications currently tend to be mainly non-authorial. The first one, dating from 1983, is Drugs and Money [42], and coincides with initial peer-reviewed publications in health science. Following a long time-lag, a Health Economics and Drugs Series started being published in 1995. Its first volume focused Alternative Drug Pricing Policies in the Americas [43], but TRIPS, globalization and patents gained special attention in the interval 1998–2005 [44–49], highlighting a strong dated focus on the category “patents”.

The WHO/HAI Project on Medicine Prices and Availability was initiated in 2001, with surveys conducted in every WHO region [50] and a focus on “price transparency”. It generated a vast number of publications in peer-reviewed literature and national and international health and development agencies. Studies may focus availability, pricing and affordability of medicines in specific countries, for specific pharmaceuticals (corticoids, opioids) or population groups (children) or combinations thereof (asthma medicine in Indian States) and have been published both as WHO or OECD technical reports and in peer-reviewed literature [51–53]. A pricing and reimbursement policy series was published on several on OECD member countries as from 2006.

WHO also published two guidelines on pharmaceutical pricing interventions. A first one was the Guidelines for price discounts of single-source pharmaceuticals, focusing negotiation as trading strategy, in 2003. In 2015, a guideline on country pharmaceutical pricing policies [27] was developed to assist national policy-makers in implementing policies to manage pharmaceutical prices.

## Discussion

To our knowledge, no scoping study on factors contributing to pricing of pharmaceuticals in health literature has been previously performed. Scoping studies on pharmaceuticals have featured orphan drugs [54] and access to medicines [55]. Two recent reviews dealing with the efficacy of international approaches to medicine price regulation and control [56] and policy options to reduce branded prescription prices [57] were not included in our scoping study timeframe, but point to an increasing interest in syntheses of interventions featuring medicine pricing.

Our analysis of pharmaceutical pricing peer-reviewed literature shows a wide thematic scope, with overall dominance of themes referring to trading strategies and regulatory approaches. Two thirds of it was produced by the academia and more than half after the year 2010. Quasi-experimental designs and country case studies predominate.

International agency publications seem to lag behind peer-reviewed literature in time frame and to hold a narrower thematic scope - with a strong focus on pricing transparency and other regulatory approaches to medicine pricing, following a transitory emphasis on patents at the end of the last century. There is also less variability in study design, with a near-complete absence of quasi-experimental and large dominance of empirical descriptive (case) studies. Nevertheless, availability in a wider number of languages and the fact that these international agency sites are references for a wider public probably turns it this into a more influential literature than peer-reviewed work.

Initial studies retrieved in our peer-reviewed literature search date from the 1980s and were published by non-health science journals indexed in health databases. This could be reflecting a gradual indexing of management, development, planning and economic papers by health databases. Within little over a decade, these disciplines seem to have been brought into the scope of health academia, with the emergence, in the early eighties and nineties, of the dedicated health policy and health economics journals publishing a substantial share of the manuscripts identified in this scoping review.

Discussion on monopoly power and disproportionate profits of the pharmaceutical industry dominated early non-health science literature on pharmaceutical prices [12], suggesting an emphasis on supply-side related market dynamics. Issues on pharma industry profits and other supply-side related market dynamics are now rarely the focus of life science literature. Monopoly power discussion is incidental and increasingly studies feature interventions to modulate prices, clustered under regulatory approaches and trading strategies.

Literature synthesis allowed us to come up with a simple schematic representation of five key dimensions for analysing pharmaceutical pricing. Common economic sense traditionally defines pricing in the setting of market dynamics [58]. Prices are the result of iterative interactions between buyers (the demand-side) and sellers (the supply side), based on characteristics of the product (the product-side), valued according to its relative differentiation in the market place [2].

The thematic clustering established in our synthesis captured the persistence of these classical market-dynamics dimensions and the contemporary emphasis on product-related market dynamics categories (generics, patent, innovation and competition). Additionally, it detected the introduction of two other more dominant and central thematic clusters – regulatory approaches and trading strategies. Much of the contemporary health academic literature on pharmaceutical prices has focused on in-depth discussions on these last two topics, a fact conveyed by the centrality of these thematic clusters. Both these clusters include interventions contributing to significant shifts in market equilibrium.

Generics are a favourite category in pharmaceutical pricing literature. They are studied mainly as competition-enhancers, central for price reductions, in studies on effects of generic entry in markets [14] or in comparisons between generic and originator prices [59].

Trading strategies had not previously collectively clustered as interventions, but much of the literature highlights their market distortion potential and relatively off-regulatory boundaries status. Firm pricing and launch strategies [60] dominate the discussion, with rebates/discounts [61] coming up with less frequency and centrality. In international agency publications, trading strategies are not much highlighted topic at this time.

Buyer-side trading strategies have gained importance in the context of access to pharmaceuticals in low and middle-income (LMIC) countries. Tendering [62] stands out as an important strategy to reduce acquisition costs of pharmaceuticals and expand healthcare coverage [63]. Differential pricing [32] – which describes differences in pricing established based on the consumer’s or country’s capacity to pay - is presented both as an option to enhance access to high-priced pharmaceuticals in LMIC and for tackling within country inequities [64]. Parallel trade - importing from countries where prices are lower – is also discussed as a strategy to enhance affordability [65], but one that can be circumvented by stricter regulation whenever deemed undesirable [66].

In the peer-reviewed literature, regulation dominates the price setting discussions relating it to impacts on pharmaceutical profit margins [67] innovation [16], competition [16], and availability of drugs [68]. Potential deleterious effects of regulation on innovation and R&D’s substantial contribution to prices are the highlights of initial health science literature pharmaceutical pricing [16]. Literature also points out the use of external reference pricing for newly-registered drugs may give rise to firms preferentially launching them in countries where pharmaceuticals achieve higher prices [69]. This leads to innovative drugs having globalized prices, based on high-income countries’ price levels. Apparently, for every new regulatory approach, a counter-approach in the form of different trading or other strategies may be come up.

On the other hand, WHO’s technical documents and reports tend to hold quite focus distinct perspectives, highlighting description of countries pricing and reimbursement policies for pharmaceuticals and price transparency as a “regulatory” tool against market information asymmetry and increased affordability.

A substantial number of case studies describing single-country regulatory policies is found both in peer-reviewed and grey literature and international agency documents, much of it stemming from WHO projects. Starting with a description of regulatory policies in Norway [70], and China [71], peer-reviewed literature initially featured mainly high-income countries. More recently a number of similar peer-reviewed studies have been published on LMIC countries [72]. OECD and WHO have, in contrast, been regularly publishing similar studies on LMIC, frequently available in non-English or even the native country languages.

Relationships between income and prices is brought up in literature on pricing differentials between high income countries (HIC) and LMIC [73, 74]. Other impacts of demand-related market dynamics on prices includes demand uncertainty [75] leading to prices being set above the expected values (the higher the uncertainty, the higher the price). Effects of prices on demand size [76], reallocation of demand [77] and demand elasticity [78], although not in the scope of our study, are also frequently discussed in literature, evidencing the importance of pharmaceutical prices both as input and output in demand-related market dynamics. The term demand is also seen in connection with demand-side regulatory measures [13, 79].

Our study has several limitations. We did not search through the articles’ reference lists and only did very focused hand-searching for grey literature and international agency publications, which may have caused us to omit important studies.

Our initial focus on academic literature was based on the assumption that it is readily accessed by healthcare managers and professionals and that scientific production in this field may be representative of a more general production. We may perhaps have underestimated production, but hopefully sizing of the thematic relevance has not been compromised. With regard to international agency publications, we acknowledge it was limited to six main sources, but that are broad enough to encompass the thematic issues of interest.

Also, contemporary academic peer-reviewed indexed literature is mostly indexed in English and this may be viewed as a limitation. However, English is the international academic language and it has also been shown that non-English literature changes final results very little, when it comes to reviews [80]. In this respect we acknowledge that WHO and OECD technical documents could have a much higher local penetration, as they may be available in various languages.

Additional limitations were having a single member of the team in charge of the initial category analysis and the fact that we did not always undertake full-reading of the articles, except when doubts arose. Whenever researchers are reasonably familiar with the theme being scoped, feasibility of studies is much enhanced when full-text reading is not undertaken. In fact, it seems to us that, in reading all the full texts, one misses the point of carrying out a scoping study and should be instead carrying out a full review.

As there is an imperative need of improvements in the conduction and reporting of scoping studies [81], all along the process of drafting this scoping study methodological concerns haunted us. In addition to being fortunately adherent to the recently published PRISMA extension for scoping reviews recommendations [82], we tried to develop a clear method for thematic scoping.

To our best knowledge, we identified thematic clusters capable of describing the literature, even though thematic clusters may not be totally free of crosscutting ideas and clusters might have had some overlap. The steps adopted to classify scoped literature may be in themselves one of the major results of the study. By means of an objective quantitative detection of categories and the creation of centrality scores, we had the opportunity to identify thematic clusters and highlight thematic dominances and their tendencies over time.

Topics which were found to be insufficiently discussed in connection with pricing in peer-reviewed literature, as measured by category centrality, include discounts/rebates, profits and price transparency. Biosimilars are emerging product category which will probably be soon be gaining space in pricing studies. Market liberalization, internet trading of pharmaceuticals and risk are notably absent categories. Risk only emerges under the recent discussion of risk-sharing and managed-entry arrangements for pharmaceutical financing [35]. An underrepresented category, which was much highlighted in non-health science literature in the 1950s and 1960s, is the ethics of pharmaceutical pricing.

A promising emerging category, linked to the theme “demand-related market dynamics”, is financing schemes (government, private insurance, pharmacy benefit managers, out-of-pocket payments). This category has been specifically mentioned in studies on Medicare Part D benefits [83], and will probably gain visibility in the scope of the sustainability of health care systems debate. Future studies featuring systematic comparisons of effects of different financing models on prices will possibly bring up very useful insights.

Recently, in the wake of the Sustainable Development Goals, constructive engagement of governments with the private sector is being recommended for the management of the global chronic disease burden [84]. Studies on partnering strategies adopted and actual effects on pharmaceutical prices and coverages will be interesting to follow. Hopefully, even though attention to the management of commercial and other vested interests remains paramount [84], truly innovative approaches to medicine pricing will come up, allowing us to develop new insights for the financing schemes category or to add new categories to the thematic cluster “trading strategies”.

## Conclusions and implications for research and practice

As an introduction to his 1986 essay on “The Political Economy of the Pharmaceutical Industry” William Comanor (1986) [12] revised the literature concerning the pharmaceutical industry. According to him, studies follow the political debate and issues examined and the stances adopted tend to reflect this.

As a polarized debate on profits, patents and innovation started being addressed by regulation, studies started focusing on the consequences of regulation. Thirty years ago, Comanor already wondered whether this debate “has set too narrow an agenda for the economic literature” or was missing the point as it fails to discuss the critical trade-offs for the development of effective public policy [12]. Since then, this literature is increasingly being indexed in health databases, has gained space in development and health international agencies and developed a growing taste for discussing interventions in the form of market regulation or trading strategies.

A greater attention to robust evidence-producing study designs for pricing interventions seems to be underway, as evidenced by the increasing presence of empirical “quasi-experimental designs” in this literature and emerging attempts at establishing guidelines for pricing interventions by the WHO [27]. Potential solutions envisaged for generating and broadening our current concept of “evidence” for public health practice and policy include identifying alternatives to the randomized controlled trials and conducting more practice-based research in low-resource settings [85].

Key factors contributing to improved empirical work include the availability of more and better data, along with advances in theoretical econometric understanding and study design [86]. Evaluating of quasi-experimental approaches (“natural experiments”) and gauging the value of systems modelling approaches that simulate the plausible effects of interventions are part of this challenge [85].

## Additional file

Additional file 1: **Table S1.** Search strategies. **Table S2.** Author affiliations in peer-reviewed literature according to decade. **Table S3.** Timeframe of study designs of selected articles on pharmaceutical pricing. 1981–2016. **Table S4.** Target country(s) or region(s) in individual studies on factors influencing pricing of pharmaceuticals (1981–2016). **Figure S1.** Articles on pharmaceutical pricing published in health science peer-reviewed journals. 1981–2016. (DOCX 67 kb)

## Data Availability

Data supporting the results reported in a published article can be found at the Mendeley Database [23].
