# Supplementary material for: Factors influencing pharmaceutical pricing - a scoping review of academic literature in health science
Source: J Pharm Policy Pract. 2019 Jun 27;12:24. doi: 10.1186/s40545-019-0183-0 (PMC6595602; doi:10.1186/s40545-019-0183-0)
Supplement: Supplementary file 1 — Table S1. Search strategies. Table S2. Author affiliations in peer-reviewed literature according to decade. Table S3. Timeframe of study designs of selected articles on pharmaceutical pricing. 1981–2016. Table S4. Target country(s) or region(s) in individual studies on factors influencing pricing of pharmaceuticals (1981–2016). Figure S1. Articles on pharmaceutical pricing published in health science peer-reviewed journals. 1981–2016. (DOCX 67 kb) [file 40545_2019_183_MOESM1_ESM.docx]

**SUPPLEMENTARY MATERIAL**

**Table S1 – Search strategies**

| **Database** | **Search strategies** |
| --- | --- |
| ***Health science databases*** | |
| Scielo (BVS) | tw:(preco OR pagamento OR precificação OR regulação OR custo) AND ti:(medicamento$) |
|  | tw:(price OR payment OR pricing OR regulation OR cost) AND ti:(medicines) |
|  | (mh:("Preparações farmacêuticas")) AND (mh:("Preço de Medicamentos" OR "Sistema de pagamento prospectivo" OR "Regulação governamental" OR "Custos" OR "análise de custo")) |
|  | (mh:("Pharmaceutical preparations")) AND (mh:("Drug price" OR "Prospective payment system" OR "Government regulation" OR "Cost" OR "cost analysis")) |
| Medline (PubMed) | ((((("price"[Title] OR "payment"[Title] OR "pricing"[Title] OR "cost" [Title] OR "regulation" [Title] OR "government regulation"[MeSH Terms]) AND (("medicines"[Title]) OR "pharmaceutical preparations"[MeSH Terms]) AND "humans"[MeSH Terms])))) |
| Scopus | ( TITLE ( ( pric*  OR  payment  OR  cost  OR  regulation ) )  AND  TITLE (  medicine*  OR  "medicines price"  OR  pharmaceut* ) ) |
| ***Grey Literature*** | |
| World Health Organization | “medicine” AND “price”  “medicine” AND “pricing”  “medicine” AND “payment”  “medicine” AND “cost”  “medicine” AND “regulation”  “pharmaceutical” AND “price”  “pharmaceutical” AND “pricing”  “pharmaceutical” AND “payment”  “pharmaceutical” AND “cost”  “pharmaceutical” AND “regulation” |
| Grey literature Database | “medicine” AND “price” (as “additional keyword”)  “medicine” AND “pricing” (as “additional keyword”)  “medicine” AND “payment” (as “all fields”)  medicine” AND “cost” (as “all fields”)  “medicine” AND “regulation” (as “all fields”)  “pharmaceutical” AND “price” (as “additional keyword”)  “pharmaceutical” AND “pricing” (as “additional keyword”)  “pharmaceutical” AND “payment” (as “additional keyword”)  “pharmaceutical” AND “cost” (as “additional keyword”)  “pharmaceutical” AND “regulation” (as “additional keyword”) |
| OECD | “medicines" or “pharmaceutical” |
| World Bank | Only one term is allowed. Topics selection: Health; pharmaceutical industry.  Title: Pharmaceutical + Search terms: Price, Pricing, Payment, Cost, Regulation  Title: Medicines + Search terms: Price, Pricing, Payment, Cost, Regulation |
| European Union | “price” AND “pharmaceutical”  “price” AND “medicine” |
| National Academies Press | “medicines price”  “pharmaceutical prices” |

**Table S2 – Author affiliations in peer-reviewed literature according to decade**

| Type of institution | 1980-1989 | 1990-1999 | 2000-2009 | ≥2010 | Total |
| --- | --- | --- | --- | --- | --- |
| Academia | 1 | 12 | 52 | 85 | 150 |
| Academia/Regulatory Agency or Government | - | - | 1 | - | 1 |
| Academia/Nonprofit organization | 1 | - | 1 | - | 2 |
| Academia/Private sector | - | 1 | - | 4 | 5 |
| Regulatory Agency or Government | - | - | 5 | 8 | 13 |
| Nonprofit organization | - | - | 2 | - | 2 |
| Private sector | - | 4 | 10 | 9 | 23 |
| NI | 1 | 1 | 2 | 4 | 8 |
| Total | 3 | 18 | 73 | 110 | 204 |

**Table S3. Timeframe of study designs of selected articles on pharmaceutical pricing. 1981-2016**

| **Study Design** | **1980-1989** | **1990-1999** | **2000-2004** | **2005-2009** | **2010-2014** | **2015-2016** | **Total** |
| --- | --- | --- | --- | --- | --- | --- | --- |
| Theoretical discursive | 2 | 3 | 4 | 5 | 11 | 9 | 34 |
| Theoretical modelling |  | 1 | 3 | 9 | 6 | 5 | 24 |
| Empirical descriptive |  | 7 | 8 | 10 | 10 | 5 | 40 |
| Empirical  quasi-experimental | 1 | 7 | 15 | 19 | 46 | 18 | 106 |
| **Total** | **3** | **17** | **30** | **43** | **72** | **37** | **204** |

**Table S4 – Target country(s) or region(s) in individual studies on factors influencing pricing of pharmaceuticals (1981-2016)**

| **Countries or Regions** | **Studies (N)** | **%** |
| --- | --- | --- |
| **Single country or region study (N=141)** | | |
| USA | 20 | 9.80 |
| EU | 10 | 4.90 |
| Germany | 9 | 4.41 |
| Canada | 8 | 3.92 |
| China | 8 | 3.92 |
| China | 8 | 3.92 |
| Europe | 6 | 2.94 |
| Norway | 5 | 2.45 |
| UK | 5 | 2.45 |
| Spain | 4 | 1.96 |
| Belgium | 3 | 1.47 |
| Brazil | 3 | 1.47 |
| Australia | 3 | 1.47 |
| Finland | 3 | 1.47 |
| France | 3 | 1.47 |
| Italy | 3 | 1.47 |
| Malaysia | 3 | 1.47 |
| Netherlands | 3 | 1.47 |
| Sweden | 3 | 1.47 |
| WHO | 3 | 1.47 |
| Bulgaria | 2 | 0.98 |
| Cyprus | 2 | 0.98 |
| Denmark | 2 | 0.98 |
| Greece | 2 | 0.98 |
| South Africa | 2 | 0.98 |
| All others* | 18 | 8.82 |
| **Comparative studies involving two countries/regions (N=5)** | | |
| Bulgaria/Romenia | 1 | 0.49 |
| EU/USA | 1 | 0.49 |
| France /UK | 1 | 0.49 |
| Italy/USA | 1 | 0.49 |
| Sweden/Czech Republic | 1 | 0.49 |
| **Multiple countries (N=22)** | | |
| Multiple countries | 21 | 10.29 |
| **Not applicable (N=36)** | | |
| Not applicable | 42 | 20.59 |
| *Croatia, Egypt, India, indonesia, Japan, Mali, Mozambique, New Zealand, Portugal, Saudi Arabia, South Korea, Sudan, Taiwan, Vietnam, Mercosur, Middle East Asia, OECD | | |

**Figure S1. Articles on pharmaceutical pricing published in health science peer-reviewed journals. 1981-2016**
